# Long-term diosmectite use does not alter the gut microbiota in adults with chronic diarrhea

**DOI:** 10.1186/s12866-022-02464-7

**Published:** 2022-02-12

**Authors:** Kévin Da Silva, Susie Guilly, Florence Thirion, Emmanuelle Le Chatelier, Nicolas Pons, Hugo Roume, Benoît Quinquis, Stanislav D. Ehrlich, Nassima Bekkat, Hélène Mathiex-Fortunet, Harry Sokol, Joël Doré

**Affiliations:** 1 Université Paris-Saclay, INRAE, 78350 Jouy-en-Josas, MGP France; 2 Ipsen, Boulogne-Billancourt, France; 3 External Expert Consultant Ipsen, Boulogne-Billancourt, France; 4 Sorbonne Université, INSERM, Centre de Recherche Saint-Antoine, CRSA, Saint Antoine Hospital, Gastroenterology department, 75012 Paris, AP-HP France; 5 Université Paris-Saclay, INRAE, Micalis Institute, 78350 Jouy-en-Josas, AgroParisTech France; 6 Paris Center for Microbiome Medicine, Fédération Hospitalo-Universitaire, 75012 Paris, France

**Keywords:** Smecta®, Diosmectite, Shotgun metagenomics, Gut microbiota, Chronic diarrhea, Long-term diosmectite use, Loose stools, Watery stools, Microbiota composition

## Abstract

**Background:**

Diosmectite, a natural colloidal clay, has been used worldwide for a number of approved indications, including the treatment of chronic functional diarrhea. Here, we used high-resolution whole metagenome shotgun sequencing to assess the impact of a 5 weeks administration of diosmectite (3 g/sachet, 3 sachets/day) on the fecal microbiota of 35 adults with functional chronic diarrhea.

**Results:**

Gut microbiota was not impacted by diosmectite administration. In particular, richness remained stable and no microbial species displayed a significant evolution. Segregating patients either by diosmectite response (non responder, early responder, late responder) or by nationality (Great-Britain or Netherlands) yielded the same results.

**Conclusion:**

We concluded that no microbiota-related physiological alterations are expected upon long-term treatment with diosmectite.

**Trial registration:**

Clinicaltrials.gov NCT03045926

**Supplementary Information:**

The online version contains supplementary material available at 10.1186/s12866-022-02464-7.

## Introduction

Diosmectite (also called Bentonite or Montmorillonite) is a natural colloidal clay, belonging to the dioctahedral smectite family extracted from monospecific geological deposits specially selected for their high quality. It presents a complex and stable crystallographic structure characterized by tetrahedral silica sheets alternating with sheets in which aluminium and magnesium are embedded in an octahedral coordination, taking the form of layers of fine sheets. Today, the active pharmaceutical ingredient is obtained through a complex, multi-step process to purify and remove impurities in order to yield an off-white, tasteless and odorless, stable, very fine powder with the status of a medicinal product. Aside from its many uses linked to its technical properties, it has been extensively used for medical purposes. Indeed, clay has been a natural material of traditional medicinal usage since prehistoric times [1]. It is currently used worldwide for a number of approved indications [2] including the treatment of acute diarrhea in children in association with oral rehydration solution, and in the symptomatic treatment of chronic functional diarrhea and pain associated with functional bowel diseases in adults. Its documented benefits derive from numerous controlled clinical trials [3–8] and meta-analyses [9]. Thanks to its leaflet structure and high plastic viscosity, diosmectite has a powerful coating property on the gastrointestinal mucosa. Its physicochemical properties likely contributed to its use as an anti-diarrheal gastro-intestinal protectant. Pharmacological studies revealed that diosmectite i) acts as mucus stabilizer and cytoprotector of the gastrointestinal mucosa against aggressive agents such as hydrochloric acid, bile acids, and other irritants [10], ii) exhibits a high adsorption capacity against enterotoxins, bacteria and virus [11], iii) decreases inflammation mediators [2], iv) reinforces intestinal mucosa barrier and restores the epithelial barrier defect induced by proinflammatory cytokines [12, 13] and v) decreases hypersensitivity to colorectal distension [14].

Diosmectite mode of action is associated with the ability of clay to adsorb water and intestinal gas and a large range of small molecules and particles. This has led to its use as a drug delivery controlled medium [15]. Its adhesion properties could also confer a potential to favor clearance of viral particles, bacteria or toxins. Diosmectite can further protect the intestinal mucosal barrier and reinforce epithelial regeneration [13, 16]. It has been demonstrated to restore normal mucosal permeability in children with gastroenteritis [12], and its anti-inflammatory and anti-diarrheal effects have been demonstrated in rats, pigs and human subjects [4, 17, 18]. More recently in a Caco-2 cells model of rotavirus infection in Ussing chambers, it has been demonstrated that diosmectite exerts an anti-diarrheal effect by inhibiting viral replication and the expression of NSP4, thereby inhibiting both ion secretion and cell damage induced by rotavirus, which could explain its clinical efficacy [19].

Chronic diarrhea is commonly associated with alterations of the microbiota, with loss of richness and functionalities such as colonization-prevention. Yet very little is known of diosmectite direct effects on the microbiota, and no information is currently available in humans.

The classical usage in gastroenterology calls for short term administrations, most commonly for a few days in acute conditions and comes with a very good safety profile [9, 20]. Yet with demands from an increasing fraction of the population suffering from Irritable Bowel Syndrome, the tendency has been to administer diosmectite in oral forms for several consecutive weeks. Clinical studies after chronic administrations of diosmectite have proven its effectiveness in patients with chronic functional diarrhea and in patients with diarrhea-predominant irritable bowel syndrome (IBS subtype D), according to the Rome II/III Classification [21–23]. In these studies, diosmectite was administrated from 2 to 8 weeks with a frequency of three times a day (TID). With increasing evidence for an efficacy in alleviating gut symptoms and transit disorders, the observed trend of use for longer durations is likely to expand.

This prompted the question of the potential impact of diosmectite on the microbiome upon long-term administration. Indeed, the gut microbiota contributes to intestinal homeostasis through direct regulation of the maintenance of the intestinal mucosa and stimulation of the immune system. Therefore, the intestinal microbiota is an integral component of the host’s physiology, with beneficial physiological effects for the host, reflecting a symbiotic crosstalk between cells of the intestinal epithelium and the resident microbiota. The understanding of the complex host-microbiota relationship and the possibility to modulate these interactions is of key importance for human health. In the present study, we used high-resolution whole metagenome shotgun sequencing to assess the impact of a 5 weeks administration of diosmectite (3 g/sachet, 3 sachets/day) on the fecal microbiota of adults with functional chronic diarrhea. Gut microbiota analysis was performed using a quantitative metagenomic approach, allowing the analysis at the gene and species level. This is the first study on the potential action of the chronic administration of diosmectite in adults with chronic functional diarrhea on the intestinal microbiota. Together with an improvement of transit parameters and full safety, we herein report on an absence of modulation of the intestinal microbiota in these conditions.

## Results

### Diosmectite treatment is efficient on diarrhea

**Fig. 1:**
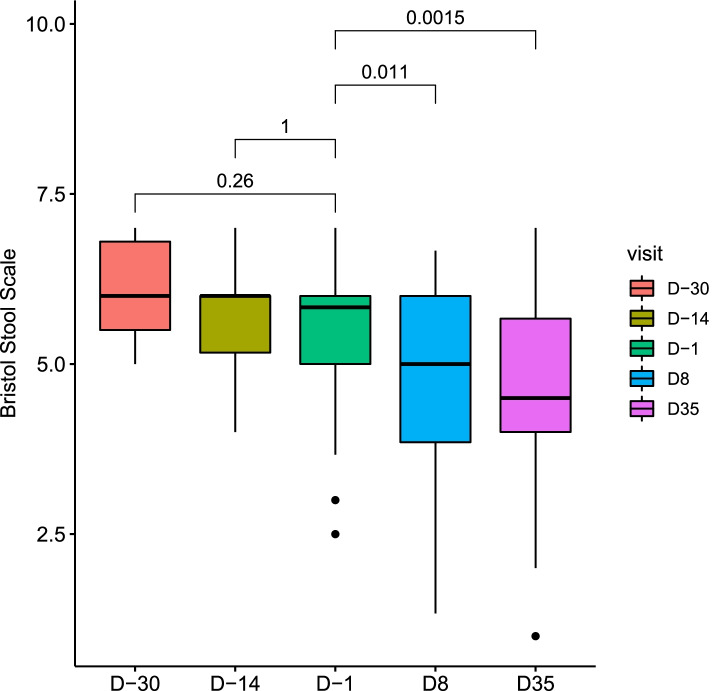
Bristol stool scale evolution according to visit. *P*-values associated with Wilcoxon signed-rank test are displayed. Boxes represent the median and interquartile ranges (IQRs) between the first and third quartiles; whiskers represent the lowest or highest values within 1.5 times IQR from the first or third quartiles

Chronic functional diarrhea defined according to the Rome IV criteria with loose or watery stools according to Bristol stool scale grade 6 and 7, occurring in at least 75% of stools for the last 3 months (with symptoms onset at least 6 months before diagnosis), with or without pain [24]. At D–30 mean Bristol stool scale (BSS) was 6.15 ± 0.7. At day D-1, 74% of individuals had diarrhea according to the inclusion criteria, and this number dropped to 40% and 49% after 8 or 35 days of treatment, respectively (*p* = 0.01 and 0.06, chi-squared test), suggesting an impact of the treatment. Accordingly, BSS decreased significantly during the treatment, going down from 5.49 ± 1.07 at D-1 to 4.69 ± 1.38 at D8 (*p* = 0.01, Wilcoxon signed-rank test) and 4.57 ± 1.39 at D35 (*p* = 1.5e-03, Wilcoxon signed-rank test) (Fig. 1). Mean stool frequency was minor than 3 per day at baseline and was not affected by the treatment (see Additional file 1, Supplementary Fig. 1), leading us to consider only BSS for treatment effect analysis in the following sections.

### Gut microbiota is stable during treatment

**Fig. 2:**
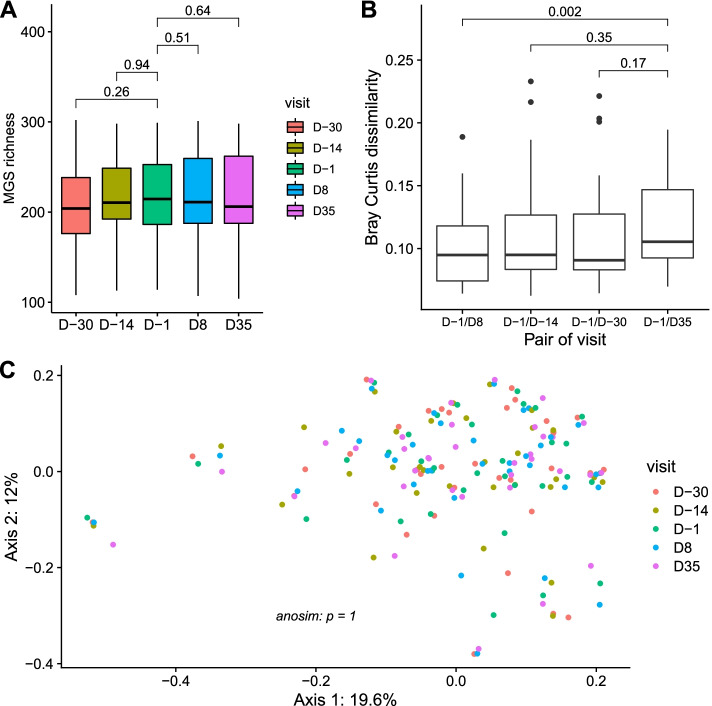
Microbiota evolution according to visit. **A** MGS richness evolution according to visit. **B** Boxplots of Bray–Curtis dissimilarity between MGS abundance at baseline (D-1) and MGS abundance at other time points (D-30, D-14, D8 or D35). *P*-values associated with Wilcoxon signed-rank test are displayed. Boxes represent the median and interquartile ranges (IQRs) between the first and third quartiles; whiskers represent the lowest or highest values within 1.5 times IQR from the first or third quartiles. **C** Principal Coordinates Analysis (PCoA) performed on Bray–Curtis dissimilarity matrix computed on MGS abundances. Patients are colored according to visit, and the analysis of similarity between different visits was computed through ANOSIM

We generated a minimum of 20-million high quality reads per sample, and mapped them onto the Integrated Gene Catalogue (IGC) [25] comprising 9.9 million genes, clustered into 1438 Metagenomic Species [26] (MGS). MGS richness (number of MGS whose abundance is strictly positive) was computed for each individual and each time point, allowing to analyze its stability over time and following the treatment (Fig. 2A). First, we analyzed the MGS richness stability before the treatment (D-30, D-14, and D-1). Wilcoxon signed-rank tests between D-30 and D-1, then D-14 and D-1 showed no significant differences (*p* = 0.26 and *p* = 0.94, respectively). Then, we analyzed the MGS richness stability during-treatment (D8 and D35). Wilcoxon signed-rank tests between the reference time point D-1 and the two on- treatment time points showed also no significant differences (*p* = 0.51 and *p* = 0.64, respectively).

Using the MGS abundance, we computed Bray–Curtis dissimilarity index between samples. We observed a decrease in similarity when the time between sampling increases (Fig. 2B). Thus, dissimilarity between D-1 and D35 is significantly higher than dissimilarity between D-1 and D8 (35 days vs 8 days, respectively, *p* = 0.002, Wilcoxon signed rank test), whereas the difference is not significant between D-1/D35 and D-1/D-30 (35 days vs 30 days, respectively, *p* = 0.17, Wilcoxon signed-rank test). Principal Coordinates Analysis on the Bray–Curtis dissimilarity index showed that the samples did not clustered according to time points (*p* = 1, ANOSIM, Fig. 2C).

**Fig. 3:**
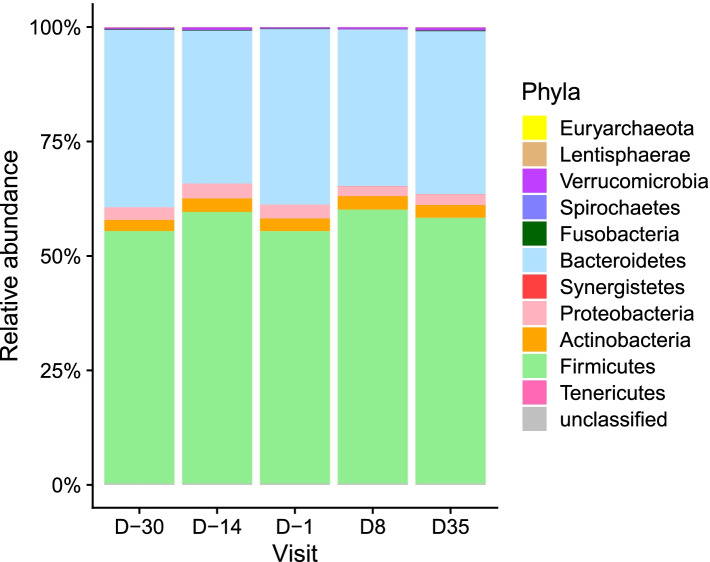
Phyla distribution evolution. Mean phyla relative abundance along the different time points

The phylum distribution remained globally constant along the timeline (Fig. 3, *p* = 1, Chi-squared test). An Area Under Curve (AUC) analysis (see Methods) revealed that *Proteobacteria* had a different evolution before and during treatment (*p*-value = 0.009, corrected p-value = 0.08, Wilcoxon signed-rank test). However, abundance of this phylum showed an alteration over time before treatment (between D-30 and D-1, *p* = 0.07, Friedman test) but not during treatment (between D-1 and D35, *p* = 0.57, Friedman test).

At lower taxonomic ranks, three families (Streptococcaceae, Eggerthellaceae, and Bifidobacteriaceae) and four genera (*Bifidocbacterium*, *Parasutterella*, *Streptococcus,* and *Turicibacter*) evolved differently before and during treatment (*p* ≤ 0.05). Friedman tests revealed that the three families and *Bifidobacterium* changed before treatment (p ≤ 0.05) whereas none of these taxa presented a significant evolution during treatment (*p* ≥ 0.1).

We further looked for diosmectite impact at the species level. Considering the 450 MGS that were present in more than 10% of samples, the AUC analysis showed that 18 MGS had a different evolution before and after treatment (*p* ≤ 0.05, Wilcoxon signed-rank test). Among them, only 4 and 5 MGS had a significantly different abundance in at least one of the three time points before treatment or during treatment, respectively (p ≤ 0.05, Friedman test). The five MGS whose abundance changed during the treatment had low prevalence at D-1 (32% ± 0.1, see Additional file 1, Supplementary Fig. 2) and accounted for only 0.3% ± 0.5 of the total microbiota. Moreover, we found the same number of MGS (*n* = 35) whose abundance changed significantly before the treatment (at D-30, D-14 or D-1, Friedman test, *p* ≤ 0.05), or during the treatment (at D-1, D8, D35, Friedman test, p ≤ 0.05), suggesting even more random variations instead of an effect of the treatment. Additionally, after correction for multiple testing only one MGS was significantly altered before the treatment, and none during the treatment.

We validated this method and confirmed that MGS were not impacted by diosmectite treatment using another approach (non-parametric tests for longitudinal data, see Supplementary Fig. 3, Additional File 1, Supplementary Table 1 in Additional File 2).

Thus, minor changes were detected in the microbiota composition during diosmectite treatment. However, since these changes affected MGS with low prevalence and were similar to changes that occurred in the microbiota before treatment, they might be the results of time fluctuations rather than diosmectite impact.

### Gut microbiota is not related to symptoms before or during treatment

Overall, we did not find any relation between BSS and gut microbiota. We correlated MGS richness with BSS at each timepoint (see Additional file 1, Supplementary Fig. 4). The association was never significant (*p* > 0.1, Spearman’s correlation) and had inconsistent direction. We then searched for MGS related to BSS before treatment (D-30, D-14, and D-1). Globally, 64 MGS (14%) were significantly correlated at one timepoint only, and their non-significant associations at other time points displayed inconsistent directions (see Additional file 1, Supplementary Fig. 5). Only one MGS (an *unclassified Clostridiales*) was significantly correlated to BSS at two different time points (D-30 and D-14, *p* ≤ 0.05, Spearman’s correlation), and none was associated at the three considered time points.

### Response to Diosmectite is not influenced by microbiota

**Fig. 4:**
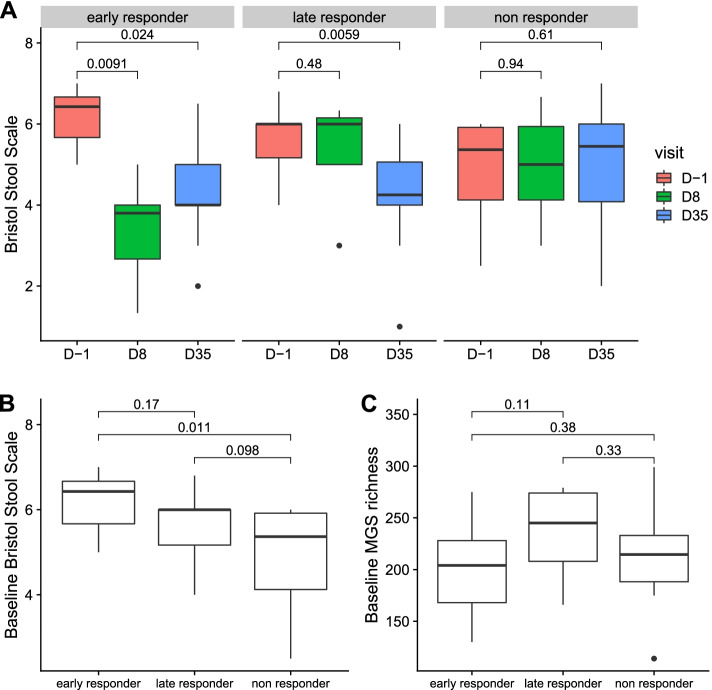
Early-, late-, and non-responders. **A** Bristol stool scale evolution across the different time points according to responder subgroups. P-values associated with Wilcoxon signed-rank test are displayed. **B** Bristol stool scale at baseline (D-1) according to responder subgroups. P-values associated with Mann–Whitney test are displayed. **C** MGS richness at baseline (D-1) according to responder subgroups. P-values associated with Mann–Whitney test are displayed. Boxes represent the median and interquartile ranges (IQRs) between the first and third quartiles; whiskers represent the lowest or highest values within 1.5 times IQR from the first or third quartiles

Using the k-means method on BSS, individuals were segregated between early responders (*n* = 9), late responders (*n* = 10) and non-responders (*n* = 14) to treatment (see Methods). Two individuals were removed from this analysis because of missing data. BSS of early responders decreased significantly at D8, those of late responders decreased significantly at D35, whereas BSS of non-responders remained stable (Fig. 4A).

The three subgroups were similar for age, sex and country of residence, but were different for BSS at baseline (Table 1 and Fig. 4B). The early responders had the highest BSS whereas the non-responders had the lowest BSS (6.2 ± 0.73 and 4.9 ± 1.2, respectively, p = 0.011, paired Wilcoxon test).

**Table 1:** Characteristics of the early responders, late responders and no responder at baseline (D-1)

	**Early responder**	**Late responder**	**Non responder**	**p early/non**	**p early/late**	**p non/late**
Number	9	10	14	-	-	-
Age (years)	41 ± 14	36 ± 13	36 ± 12	0.31	0.44	0.75
Sex (Female %)	22	40	50	0.37	0.74	0.94
Country (GBR %)	89	40	71	0.64	0.084	0.26
Bristol stool scale	6.2 ± 0.73	5.6 ± 0.78	4.9 ± 1.2	0.011	0.17	0.098
MGS richness	200 ± 46	240 ± 43	220 ± 48	0.38	0.11	0.33

*P*-value correspond to Mann–Whitney test for quantitative variables (age, Bristol scale, MGS richness), and for Chi-squared test for categorical variables (sex, country expressed as Great Britain percentage). Quantitative values are displayed as mean ± standard deviation

At baseline, MGS richness was higher in late responder as compared to early responder (240 ± 43 and 200 ± 46, respectively), but this difference was not significant (*p* = 0.11, Wilcoxon test, Fig. 4C), possibly due to the low number of individuals. A Principal Component Analysis (PCA) based on MGS abundance at baseline did not reveal a difference in global composition (*p* > 0.05, ANOVA on the first two components). Performing Kruskal–Wallis test on the 450 MGS at D-30, D-14 or D-1, we found 8 MGS (1.8%) that were consistently contrasted between the three subgroups at the different time points. Out of these 8 MGS, 7 were more abundant in the late-responder group when compared to both early-responder and non-responder groups (p ≤ 0.05, post-hoc Dunn test), which was consistent with the highest richness observed in the late-responder group. Following the same procedure as above based on AUC, there were respectively 0, 2, and 2 MGS that significantly changed during the treatment in the early responders group, the late responders group or the non-responders group (see Additional file 1, Supplementary Fig. 6). Given the low number of MGS changing during the treatment in different subgroups, we could conclude that diosmectite did not alter the gut microbiota in the individuals, no matter how responsive they were to the treatment. Similar analysis on individuals segregated by country of residence also showed no alteration of the gut microbiota (see Additional file 1, Supplementary Analysis, Supplementary Table 2, Supplementary Figs. 7–8-9).

## Discussion

The current use of diosmectite being for chronic as well as acute diarrhea, the potential modification of the gut microbiota induced by a long-term administration, clearly warranted the assessment of its impact on the intestinal microbiome.

Based on Bristol stool scale assessment, we herein confirmed its clinical benefit in subjects with chronic diarrhea, with a reduction in overall BSS. The added observation made here is that the impact was all the more important when severity at baseline was more marked.

In concordance with the absence of adverse effects in terms of symptomatology, we observed a complete lack of impact on the microbiota, with no sign of alteration at the highest resolution attained by a complete shotgun sequencing-based metagenomic gene scan. No microbiota related adverse effects were to be expected, such as alteration of metabolism and ensuing modulations of intestinal permeability, inflammation or oxidative stress. This can be seen as contrasting with chronic treatments such as those used for type-2 diabetes or neuropsychological disorders. In such cases, the drug-microbes interactions are at stake and can markedly alter the microbiota [27, 28].

In a former large population study, Vieira-Silva et al. [29] showed a relationship between BSS and microbiota richness based on quantitative metagenomics assessment, confirming results obtained through 16S rDNA profiling study [30]. This was not observed in the present study but this could be due to the inclusion of long-term IBS patients with chronic functional diarrhea defined as “Loose (mushy) or watery stools”, i.e. a rather homogeneous phenotype, all having elevated baseline BSS (6 to 7 for at least 75% of stool for the past 3 months). In this study, the diarrhea was not marked, as the number of stools was normal and the mean BSS was 5.49 ± 1.07 at baseline, and lower i.e. respectively 4.9 ± 1.2 in non-responder (*n* = 14) and 6.2 ± 0.73 in responders at D8 (*n* = 9). Considering the rather low number of patients and their fairly homogeneous and narrow range of BSS compared for example to a general population, we did not observe a correlation between BSS and microbiota parameters. We therefore cannot exclude the absence of a link between the microbiome and the symptoms or their modulation in the population under study. In this context, we acknowledge that an untreated control arm would have been useful, but it was not included in the design selected for the primary outcome of which this study is ancillary.

Considering its importance in the microbiota composition, another limit of the study is the absence of data related to diet apart from the exclusion criteria (artificial feeding or subjects eating shellfish more than two times a week, see Methods).

Further in-depth investigations should include a finer assessment of the impact on microbiome gene expression (metatranscriptome) or metabolome to assess whether in spite of a lack of compositional change, the microbiota might be modulated in its functions, especially because of the ability of clay to absorb a large range of small molecules. Nonetheless, the overall lack of impact on the microbiota should be regarded as a rather positive observation suggesting diosmectite can be administered for rather long periods of time (up to 5 weeks in this study) without causing any risk of microbiota-mediated gut symptoms, such as bowel distension or intestinal inflammation.

## Conclusion

Diosmectite (Smecta®) has a well-documented efficacy and safety in acute and chronic transit disorders but its long-term administration in chronic diarrhea prompted this study to explore its potential impact on the intestinal microbiota. Over a 5 weeks administration, diosmectite did improve transit on the basis of BSS and yet it did not alter the microbiota composition based on high resolution metagenomics. Hence, consistent with the absence of adverse events, no microbiota-related physiological alterations are expected upon long-term treatment with diosmectite.

## Materials and methods

### Study population

Thirty-five adults subjects were enrolled, 20 males and 15 females, whose country of residence was Netherlands (NLD; *n* = 12) or Great Britain (GRB; *n* = 23). The inclusion criteria were: male or female, between 18 and 60 years old, BMI between 19 and 32 kg/m^2^, minimum body weight of 50 kg. They had functional chronic diarrhea defined according to the Rome IV criteria with loose or watery stools according to Bristol stool scale (BSS) grade 6 and 7, occurring in at least 75% of stools for the last 3 months (with symptoms onset at least 6 months before diagnosis), with or without pain [24]. Subjects with history of suspected organic or drug induced cause to chronic diarrhea were excluded as well as antibiotic, metformin and/or Proton Pump Inhibitor intake within the month prior to baseline visit or during the study. The inclusion took place from 24 August 2016 to 9 May 2017, in the Netherlands (PRA Healthy Sciences, Groningen, 9728NZ), and in the United Kingdom (MAC Clinical Resarch Limited, Manchester, M13 9NQ).

### Treatment

The subjects were treated with diosmectite (Smecta® 3 g/sachet, powder for oral suspension, Ipsen, France). It consisted in three sachets per day (TID), administrated at morning, noon, and evening. Subjects were instructed to take diosmectite fasting and at least one hour before meal, except for breakfast at least ½ hour before.

### Study design

This study was conducted in compliance with the protocol, in accordance with the International Conference on Harmonisation Good Clinical Practice (CPMP/ICH 135/35) together with such other good clinical practice requirements and the ethical principles that have their origin in the Declaration of Helsinki, as well as with all currently applicable laws and regulations of the country where the study was conducted. Informed consent was obtained from all subjects during the screening period. The trial was registered with Clinicaltrials.gov, number NCT03045926 on the 08/02/2017 (https://clinicaltrials.gov/ct2/show/NCT03045926).

The clinical trial was a prospective, open label, non-comparative, multi-center, international study with chronic treatment of diosmectite (Smecta®, 3 g) TID over 5 weeks, whose first purpose was to assess the level of elemental impurities (e.g. lead, arsenic, cadmium) in blood and urine samples after chronic administration of diosmectite. The aim of this ancillary study was to assess the bowel microbiota composition, stools consistency and frequency after chronic administration of diosmectite in subjects with chronic functional diarrhea.

Exclusion criteria included:

- Artificial feeding;
- Subjects eating shellfish (crustaceans, mollusks) more than 2 times a week;
- Antibiotic agent intake within the month prior to baseline visit (Day -1).
- Risk of antibiotic treatment course during the study.
- Need for metformin and or proton pump inhibitors (PPI) intake within the month prior to baseline or during the study.

After a screening period of up to 6 weeks including a baseline assessment, each subject was dosed with diosmectite TID over 5 weeks (Day 1 to Day 35). Feces samples were collected at screening phase (D-30, D-14), at baseline visit (D-1), and over the treatment period (D8, D35). Collection was performed using a kit provided by INRAE using a stabilizing solution (RNAlater®; ThermoFisher Scientific, Waltham, US) (SOP05_V2 from the International Human Microbiome Standards, IHMS) [31] allowing samples to be preserved within 24 h to 7 days at room temperature before to be handled by laboratory.

Consistency of stools (recorded and rated according to BSS) and frequency were assessed over 24 h preceding the stool sample by the subject.

Among the 35 participants, five were missing one or several samples (maximum missing samples: 3 at D-14), leading to a total of 170 samples available for the analysis.

### DNA extraction and sequencing

Fecal DNA was extracted following the SOP07_V2 from IHMS procedure [31, 32]. The DNA preparation was subjected to quality control using Qubit Fluorometric Quantitation (ThermoFisher Scientific, Waltham, US) and qualified using DNA size profiling on a Fragment Analyzer (Agilent Technologies, Santa Clara, US). 3 µg of high molecular weight DNA (> 10 kbp) was used to build the library. Shearing of DNA into fragments of approximately 150 bp was performed using an ultrasonicator (Covaris, Woburn, US) and DNA fragment library construction was performed using the Ion Plus Fragment Library and Ion Xpress Barcode Adapters Kits (ThermoFisher Scientific, Waltham, US). Purified and amplified DNA fragment libraries were sequenced using the Ion Proton Sequencer (ThermoFisher Scientific, Waltham, US), with a minimum of 20 million high-quality reads of 150 bp (in average) generated per library. We generated a mean of 22.3 million (± 0.8 million) reads per sample.

### Reads mapping

Read cleaning, filtering and mapping were performed with the METEOR software suite (parameters: -c smart_shared_reads) [33] that relies on Bowtie2 for read mapping [34]. First, quality control was performed with AlienTrimmer (parameters: -k 10 –l 45 –m 5 –p 40) [35]: sequencing adapters were removed and low quality reads were trimmed or discarded. Then, reads mapped to the human genome (identity ≥ 90%) were also discarded. Food genome traces were removed by the same method. Remaining reads were mapped to the Integrated Gene Catalogue (IGC) [25], comprising 9.9 million of genes (default parameters of Bowtie2). Uniquely mapped reads (reads mapped to a unique gene in the catalogue) were attributed to their corresponding genes. The shared reads (reads that mapped with the same alignment score to multiple genes in the catalogue) were attributed according to the ratio of their unique mapping counts of the captured genes. The resulting count table was further processed using the MetaOMineR R package v1.31 [36]. Downsizing at 14.5 million mapped reads was performed to take into account differences in sequencing depth and in mapping rate across samples. Then, the downsized matrix was normalized according to gene length and transformed into a frequency matrix (FPKM normalization).

### Metagenomic species

The IGC has been previously clustered into 1438 MetaGenomic Species (MGS; clusters of > 500 co-abundant genes belonging to the same microbial species) [26]. Taxonomical annotation of MGS was performed using an in-house pipeline. First, all genes are aligned on public databases (ncbi, wgs) [37] using blastn (version 2.7.1, task = megablast, word_size = 16) [38]. The 20 best hits for each gene were kept. A species-level assignment was given if > 50% of the genes matched the RefSeq reference genome of a given species, with a mean identity ≥ 95% and mean gene length coverage ≥ 90%. The remaining MGS were assigned to a higher taxonomic level (genus to superkingdom), if > 50% of their genes had the same annotation. Relative abundance of an MGS was computed as the mean abundance of its 50 ‘marker’ genes (that is, the genes that correlate the most altogether in terms of abundance). If less than 10% of ‘marker’ genes were seen in a sample, the abundance of the MGS was set to 0. Relative abundances at higher taxonomical ranks were computed as the sum of the MGS that belong to a given taxa.

### Gene and MGS richness

Gene richness was computed as the sum of genes whose abundance was strictly positive after downsizing. There were 595,000 ± 131,000 genes in each sample (mean ± sd). Similarly, MGS richness was computed as the sum of the MGS present in one sample. There were 215 ± 49 MGS in each sample. Detailed information about gene and MGS richness in each individual at each time point is available in the Additional file 2 (Supplementary Table 3–4).

### Statistical analysis

All statistical analyses and graphs were performed with R software (v3.6.0) [39]. Differential analysis between features (richness, MGS, higher taxonomic rank, etc.) were performed using either: (1) Wilcoxon signed-rank test for comparison between two time points; (2) Friedman test for comparison between more than two time points followed by post-hoc Nemenyi test; (3) Mann–Whitney test for comparison between two groups at one time point; (4) Kruskal–Wallis test for comparison between more than two groups at one time point. Correlations between variables (either metagenomic variables or clinical variables) were performed using Spearman’s correlations. All p-values were adjusted for multiple testing with the Benjamini–Hochberg Procedure. Unless stated otherwise, an adjusted p-value is considered significant if inferior to 0.1; a non-adjusted p-value is significant if inferior to 0.05.

Bray–Curtis dissimilarity was computed on the log-10 transformed MGS abundance table with the package *vegan* v2.5.7 [40]. Principal Coordinates Analysis was performed on the Bray–Curtis dissimilarity with the package *ade4* v1.7.16 [41]. Analysis of similarity (ANOSIM) between groups was performed with the package *vegan* [40]*.*

Effect size was computed using the Cliff’s Delta with the R package ‘effsize’ [42]. This measure gives an information similar to log-fold change but is comprised between -1 and 1 (0: no effect; + 1 or -1: large effect). Magnitude of the effect size d is assessed in Romano et al.﻿ [43] as negligible if |d|< 0.147, small if |d|< 0.33, medium if |d|< 0.474, and large otherwise.

Individuals were segregated based on k-means method, a classification method used to create groups of individuals without prior knowledge of number of classes. Individuals are aggregated around K mean “centers” so the groups are composed of the most similar individuals. Here, several K were tested and K = 3 (stratification into three groups) was selected according to the relevance of the groups obtained, confirmed by statistical analysis. The input given to the K-means algorithm were the delta values of the Bristol stool scores at D8, D35 and D125, using D-1 as the reference.

### Area Under Curve

We computed the Area Under Curve (AUC) to assess the evolution of an MGS (or higher taxonomic rank) between an initial point ${T}_{initial}$ and a final point ${T}_{final}$, a methodology that has already been used in other studies [44, 45]. For each point $T$ between ${T}_{initial}$ and ${T}_{final}$, each individual $I$ and each MGS $M$ we computed the log10 fold change between the abundance of $M$ at $T$ and its abundance at ${T}_{initial}$ (D-30 when considering points before the treatment; D-1 when considering points during the treatment). Then we computed the AUC of $M$ based on the log10 fold change: if the abundance of $M$ is minimal at ${T}_{initial}$, $M$ increases and the AUC will be positive; if the abundance of $M$ is maximal at ${T}_{initial}$, $M$ decreased and the AUC will be negative. If $M$ is stable along all considered time points, the AUC will be equal to 0.

Results obtained with the AUC were then confirmed with the function *ld.f1* from the package *nparLD* v2.1 [46] specific of longitudinal data for one homogeneous group of subjects.

## Supplementary Information

**Additional file 1.**

**Additional file 2.** 

## Data Availability

Datasets that were generated and analyzed for the current study will be available on the European Nucleotide Archive (ENA) in EBI repository, with the accession numbers PRJEB43454 (https://www.ebi.ac.uk/ena/browser/view/PRJEB43454), after manuscript acceptation.

## Abbreviations

ANOSIM: Analysis of similarity
IBS: Irritable Bowel Syndrome
TID: Three times a day
BSS: Bristol Stool Scale
IGC: Integrated Gene Catalogue
MGS: Metagenomic Species
AUC: Area Under Curve
GBR: Great-Britain
NLD: Netherlands
PCA: Principal Component Analysis
PCoA: Principal Coordinates Analysis
INRAE: Institut National de Recherche pour l’agriculture l’alimentation et l’environnement
IHMS: International Human Microbiome Standards
FPKM: Fragments Per Kilobase Million
