# Supplementary material for: Long-term diosmectite use does not alter the gut microbiota in adults with chronic diarrhea
Source: BMC Microbiol. 2022 Feb 12;22:54. doi: 10.1186/s12866-022-02464-7 (PMC8840705; doi:10.1186/s12866-022-02464-7)
Supplement: Supplementary file 1 — Additional file 1. [file 12866_2022_2464_MOESM1_ESM.docx]

**Supplementary data**

**Additional file details**

File name: Additional file 1.

Format: .docx.

Title: Supplementary data

Description:

- Supplementary analysis (impact of nationality);
- Supplementary Figures (1-9);
- Supplementary Table 2.

File name: Additional file 2.

Format: .docx

Title: CONSORT Checklist

Description: CONSORT Checklist along with the location of each item in the main manuscript.

File name: Additional file 3.

Format: .xls.

Title: Supplementary tables

Description:

- Supplementary Table 1
- Supplementary Table 3
- Supplementary Table 4

**Supplementary analysis**

***Response to Diosmectite is not influenced by country of residence***

Among the 35 individuals, 23 were living in Great Britain (GBR) and 12 in the Netherlands (NLD). Individuals from GBR were older than individuals from NLD (40 ± 12 vs 30 ± 12 years old, p = 0.032, Wilcoxon test) and had more severe diarrhea (Bristol Stool Scale (BSS) 5.8 ± 0.77 vs 4.8 ± 1.3, p = 0.031, Wilcoxon test, see Supplementary Table 1). Accordingly, GBR had an ‘early responder’ profile in BSS evolution, while NLD had a ‘late responder’ profile (Supplementary Figure 6). Microbiota at baseline was slightly different between GBR and NLD, since the 3^rd^ component of the PCA on MGS, accounting for 5.5% of variability, was significantly related to country of origin (R² = 0.14, p = 0.03, ANOVA, Supplementary Figure 7). Despite these differences at baseline, research of treatment impact on each of these subgroups yielded only 3 and 2 MGS (out of 450) significantly changing during the course of the treatment (Supplementary Figure 8). The low number of MGS show the absence of diosmectite impact in subgroups based on country of residence.

**Supplementary Figures**

**
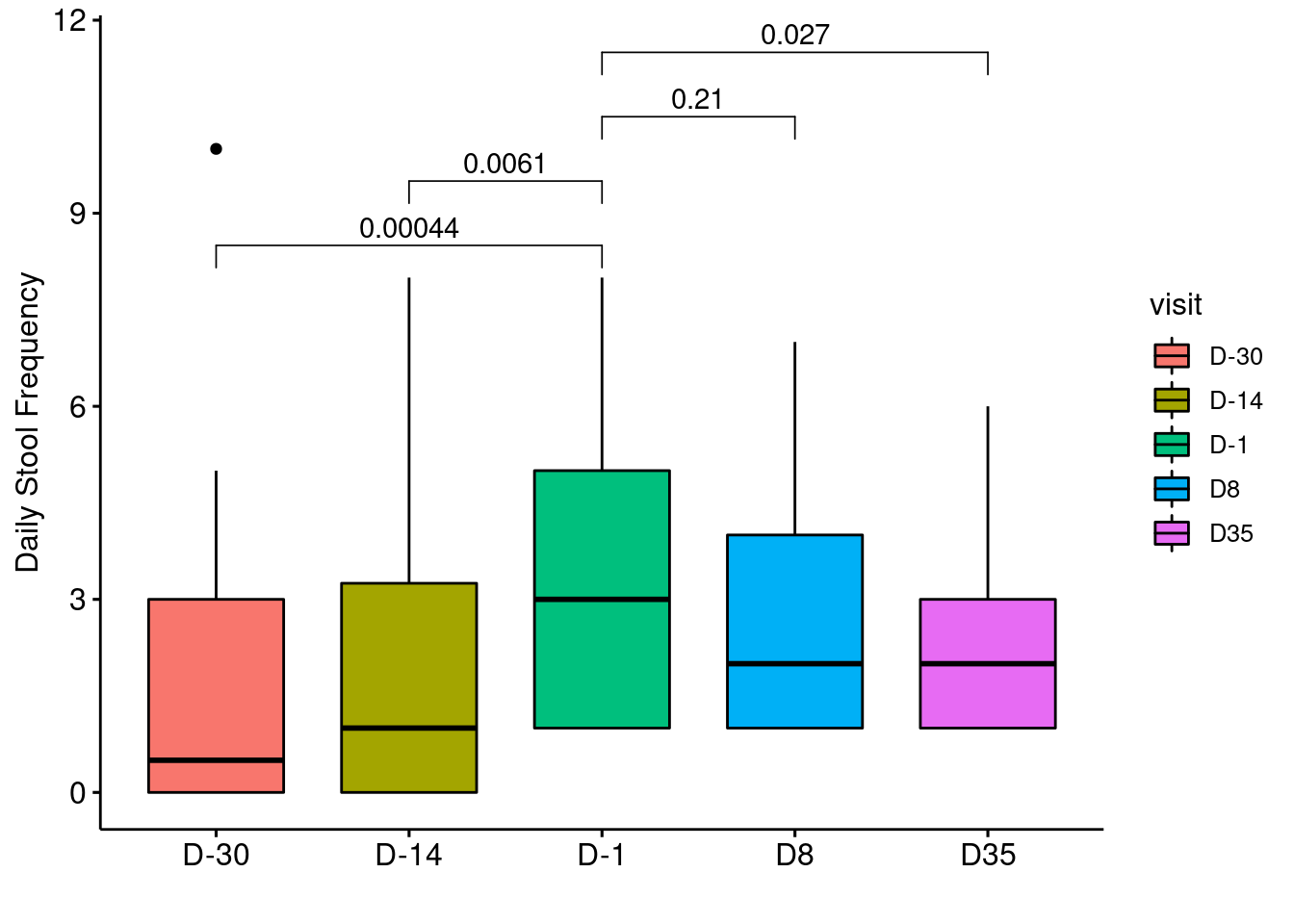
**

**Supplementary Figure 1:** Daily stool frequency evolution according to visit. P-values associated with Wilcoxon signed-rank test are displayed. Boxes represent the median and interquartile ranges (IQRs) between the first and third quartiles; whiskers represent the lowest or highest values within 1.5 times IQR from the first or third quartiles.


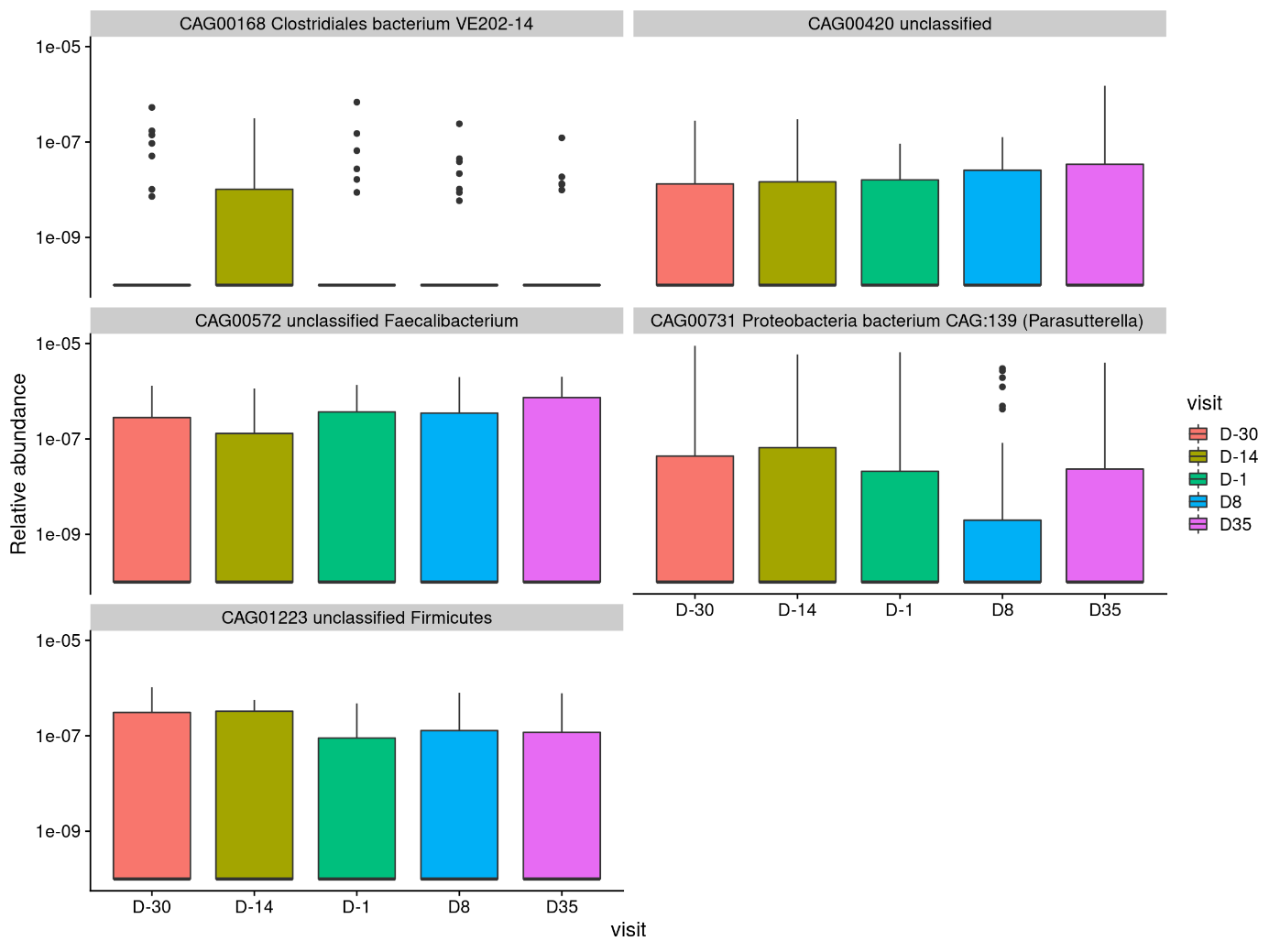


**Supplementary Figure 2:** Log-transformed relative abundance along the different time points of the 5 MGS whose AUC was significantly different before and during the treatment, and whose abundance was significantly different in at least one of the three points D-1, D8 and D35. Boxes represent the median and interquartile ranges (IQRs) between the first and third quartiles; whiskers represent the lowest or highest values within 1.5 times IQR from the first or third quartiles.


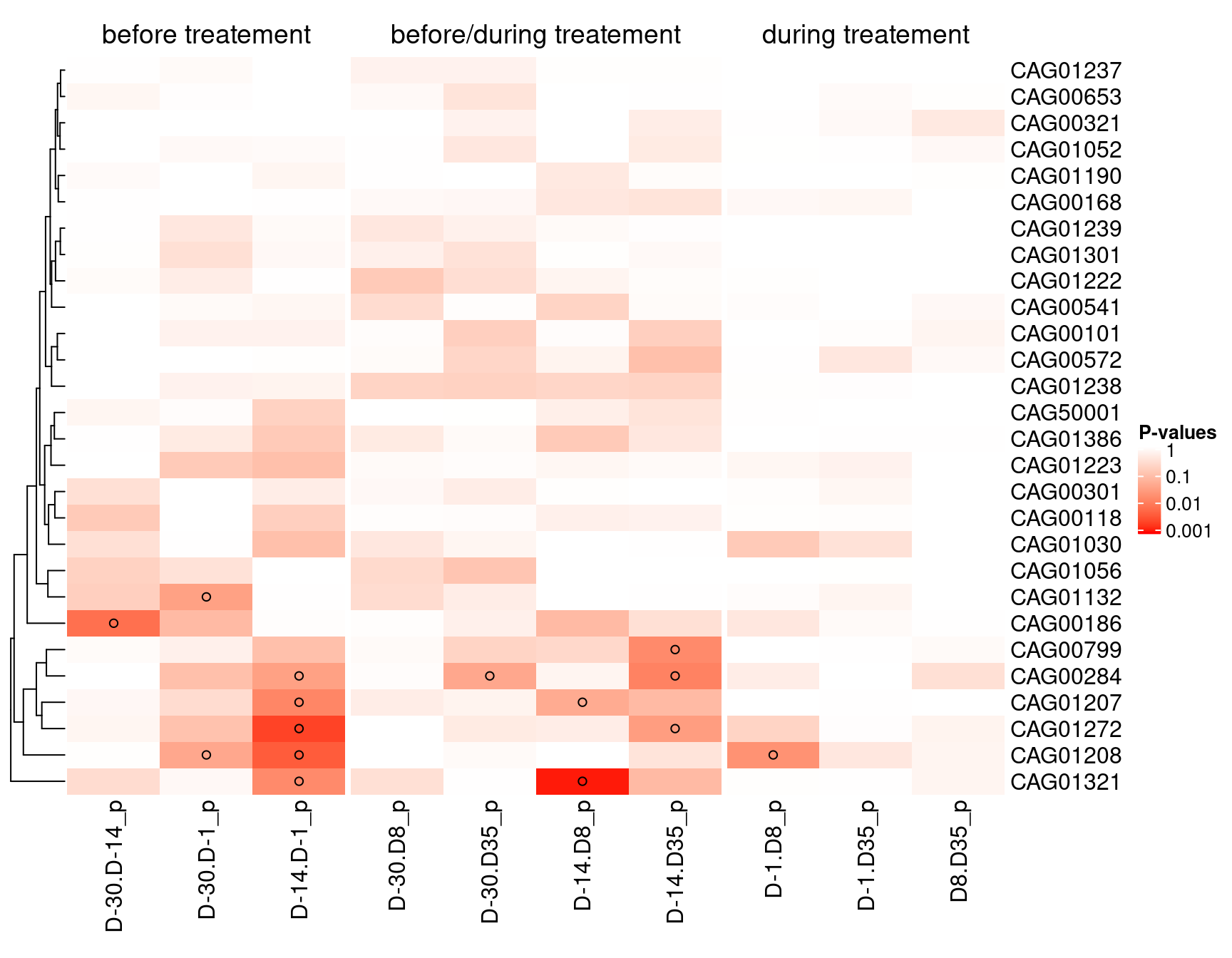


**Supplementary Figure 3:** Heatmap of p-values obtained with the post-hoc Nemenyi tests between two visits, considering MGS whose abundance changed significantly over time (non-parametric tests for longitudinal data, p ≤ 0.05). Circles highlight p-values under the threshold of 0.05. The heatmap is divided into three parts: both visits took place before diosmectite treatment (left), both visits took place during diosmectite treatment (right), or one visit was before and one visit was during treatment (middle).


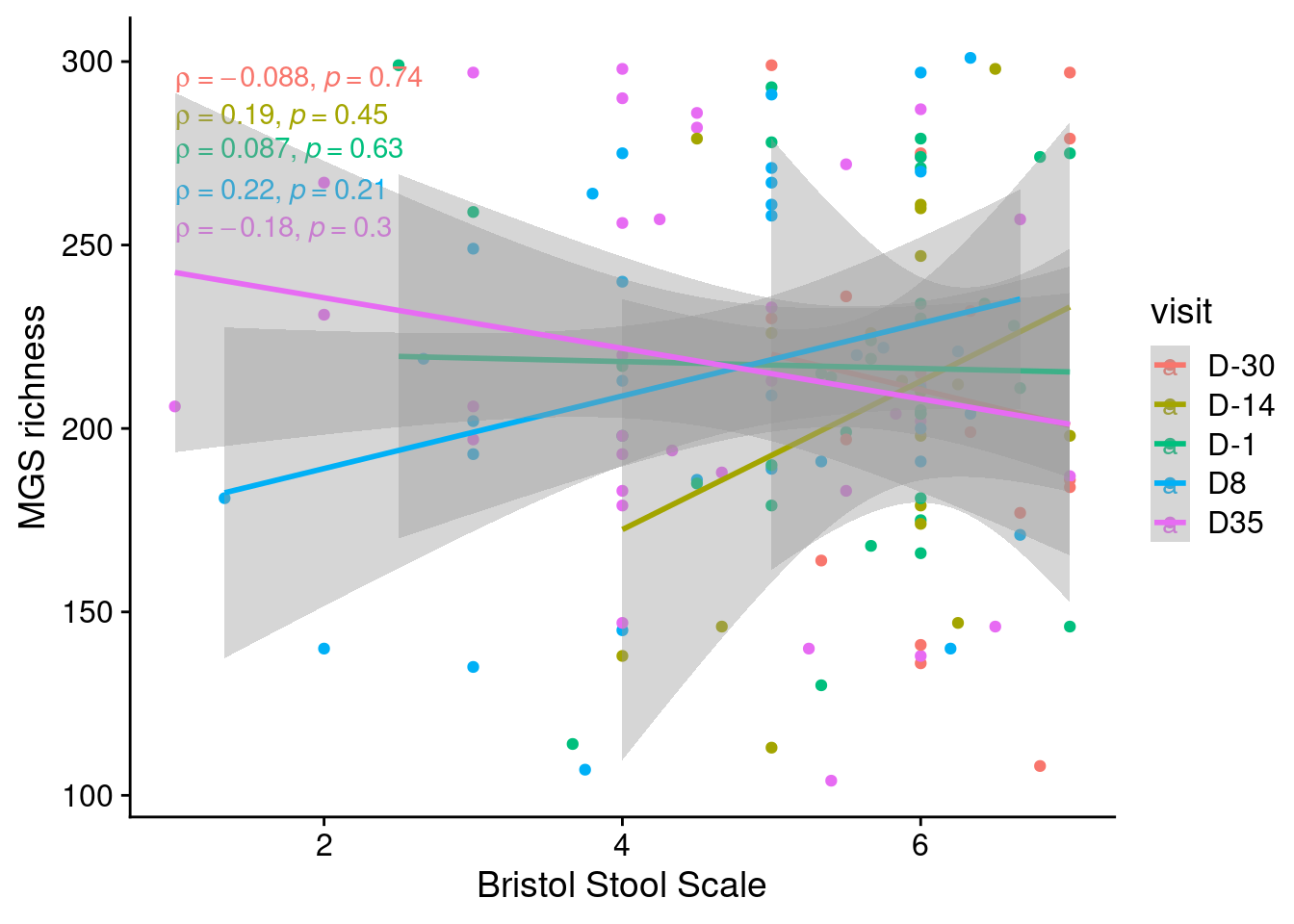


**Supplementary Figure 4:** Relation between MGS richness and BSS at the different time points. Spearman’s rho coefficients along with their associated p-value are displayed.


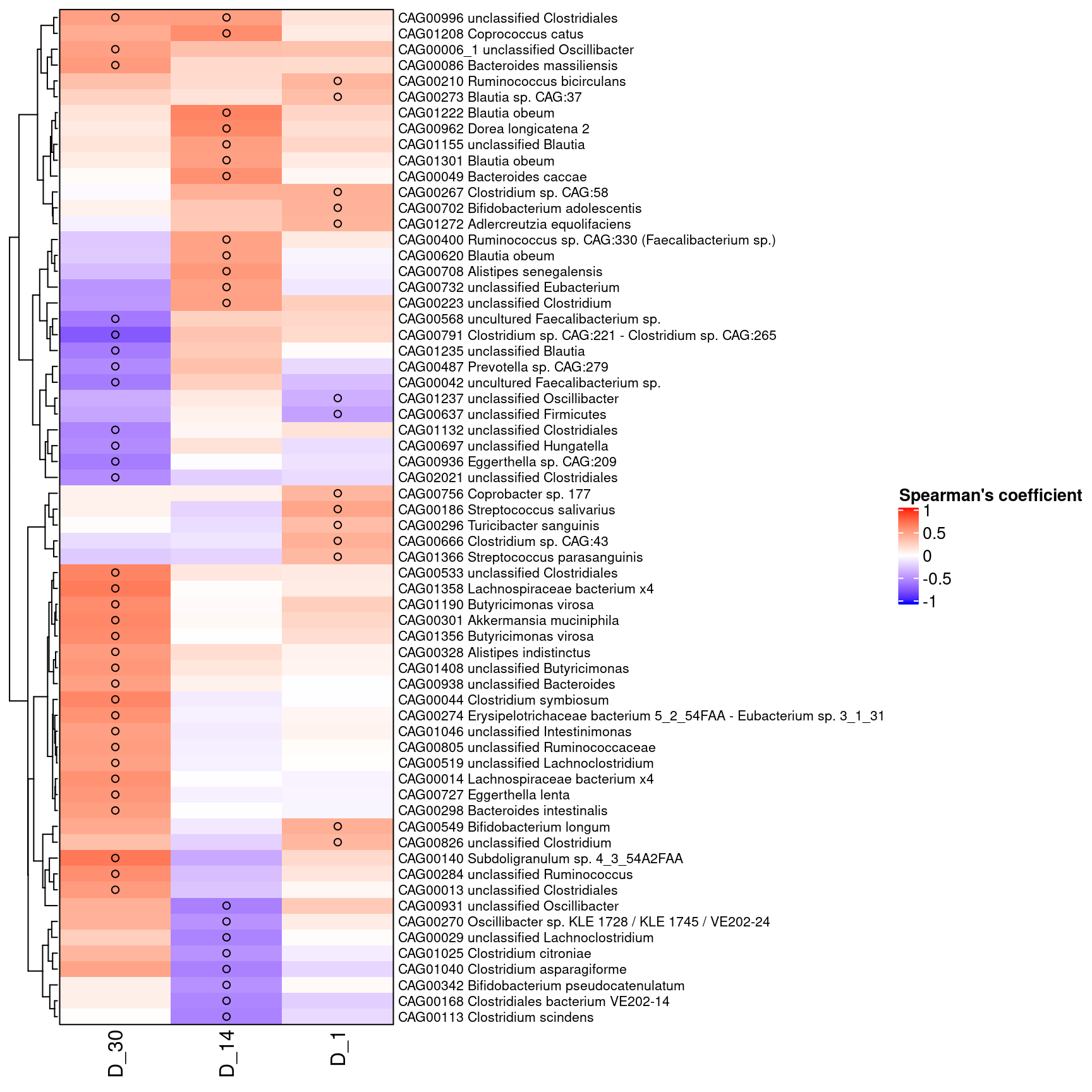


**Supplementary Figure 5**: Spearman’s correlation between MGS and BSS before treatment (at D-30, D-14, and D-1). MGS with at least one significant correlation (p ≤ 0.05) at one of the time points are displayed. Black circles represent significant relations.


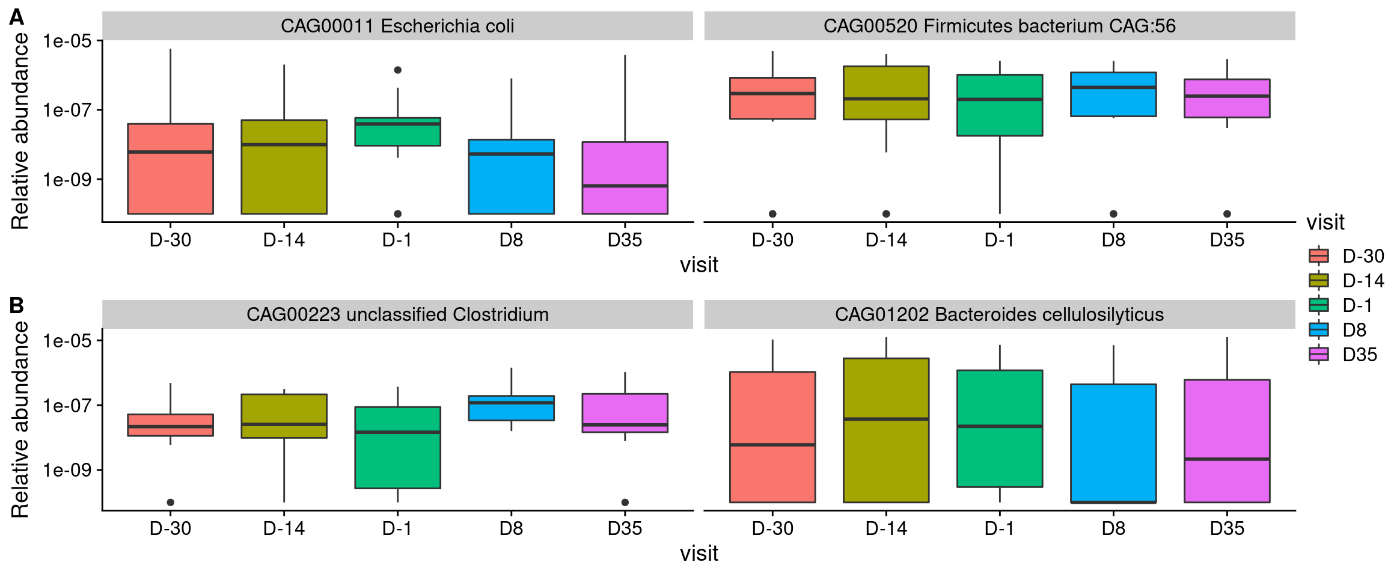


**Supplementary Figure 6:** Log-transformed abundance along the different time points of the MGS whose AUC was significantly different before and during the treatment, and whose abundance was significantly different in at least one of the three points D-1, D8 and D35 in (A) the late responder or in (B) the non responder. Boxes represent the median and interquartile ranges (IQRs) between the first and third quartiles; whiskers represent the lowest or highest values within 1.5 times IQR from the first or third quartiles.


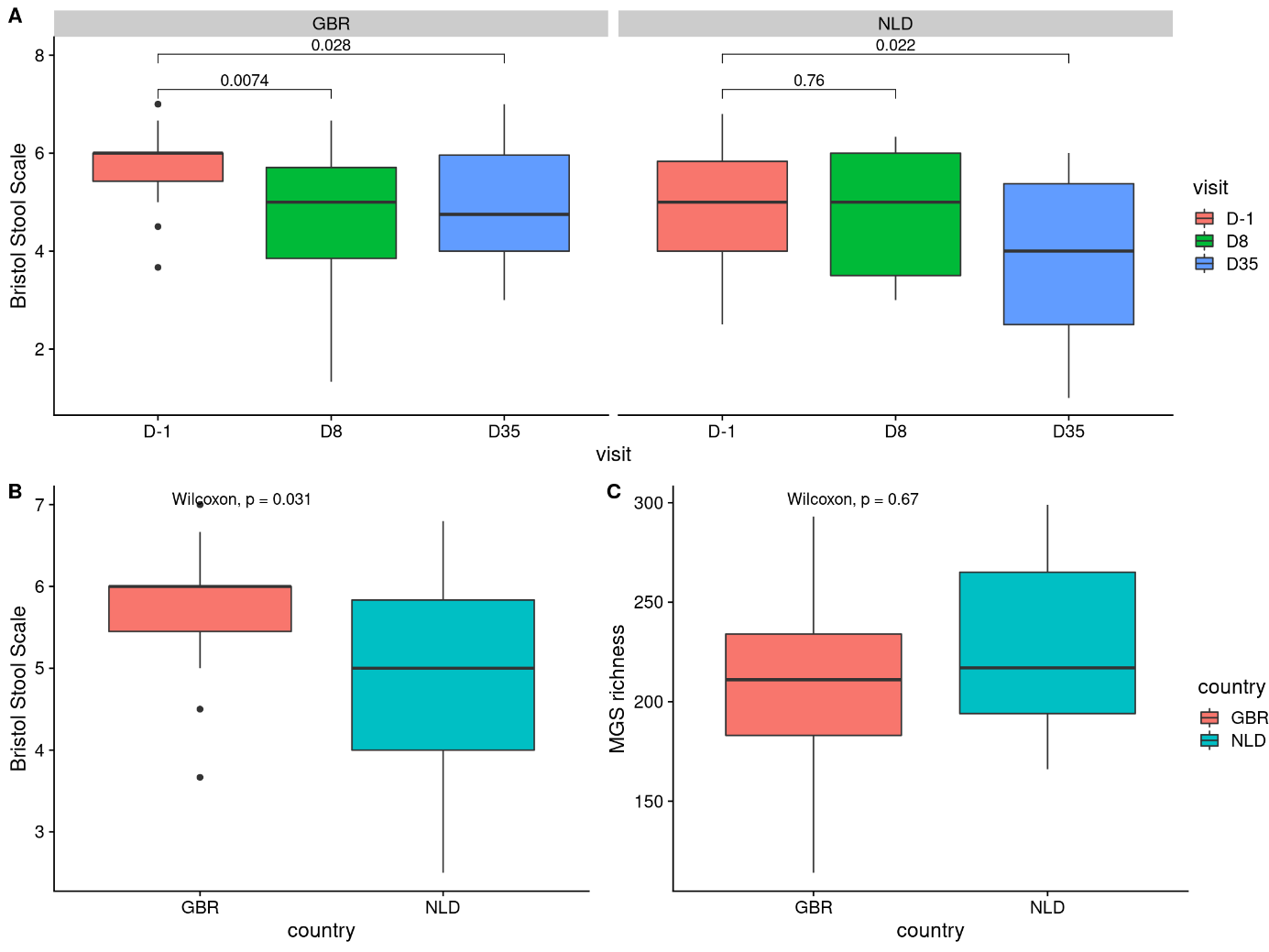


**Supplementary figure 7:** (A) Boxplots of the Bristol stool score according to visit time, in GBR only (left) or in NLD only (right). (B) Boxplots of the Bristol score at baseline in GBR and NLD. (C) MGS richness at baseline in GBR and NLD. Boxes represent the median and interquartile ranges (IQRs) between the first and third quartiles; whiskers represent the lowest or highest values within 1.5 times IQR from the first or third quartiles. *GBR = Great-Britain; NLD = Netherlands*

*
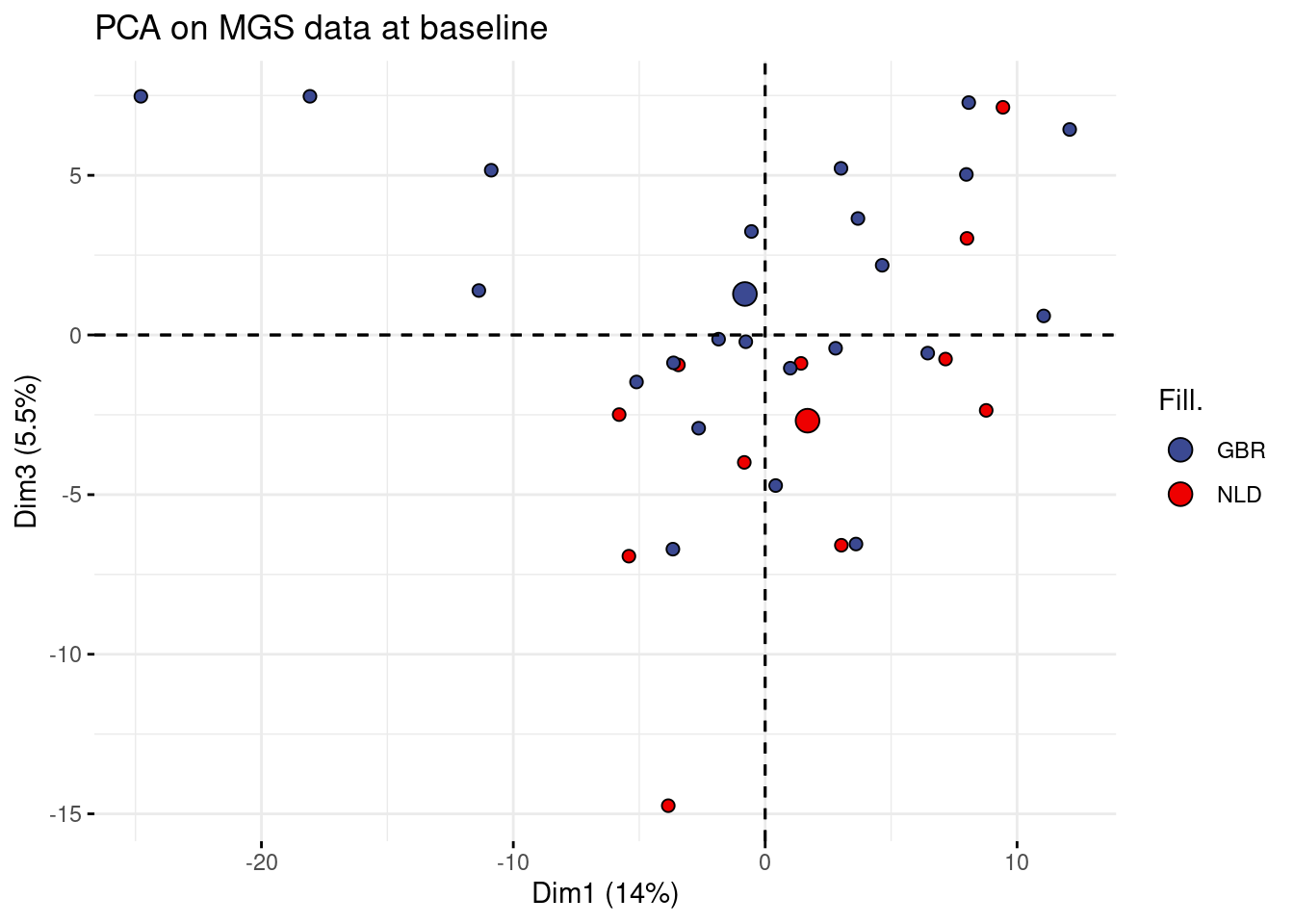
*

**Supplementary Figure 8:** Principal Component Analysis (PCA) on the 450 MGS present in at least 10% of individuals. Individuals are projected on the 1^st^ and 3^rd^ dimensions and coloured according to their country of residence. Larger circles are centroids (average value) of each group.


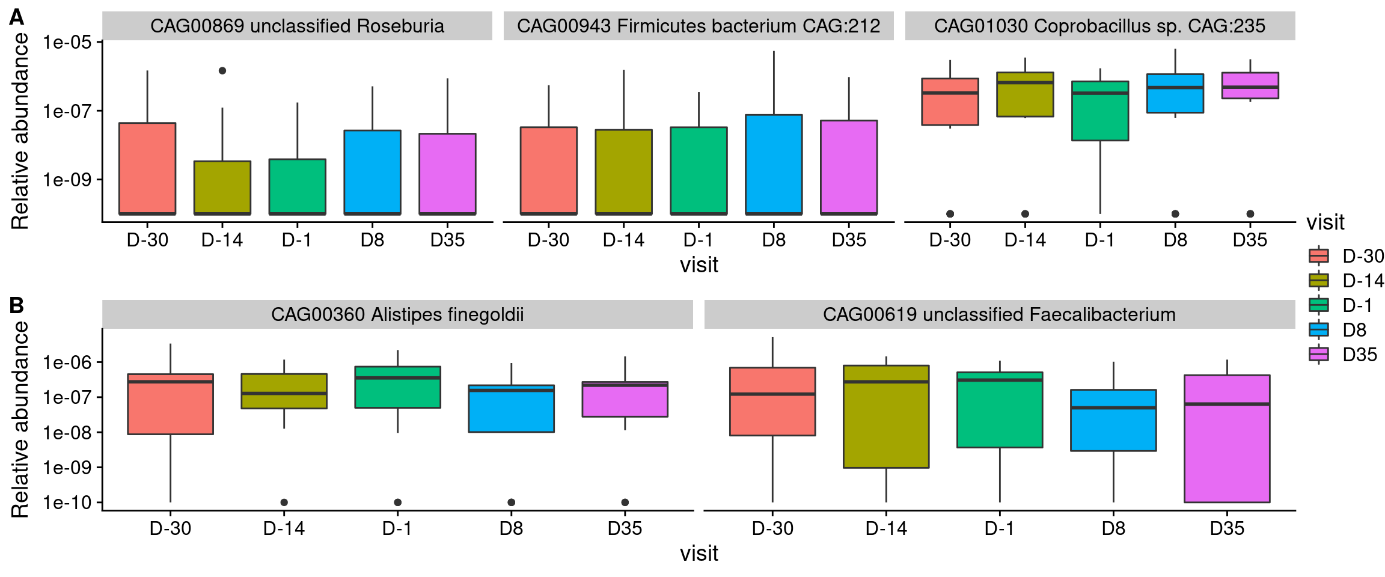


**Supplementary Figure 9:** Boxplots of MGS changing during the course of the treatment (A) in GBR and (B) in NLD. Boxes represent the median and interquartile ranges (IQRs) between the first and third quartiles; whiskers represent the lowest or highest values within 1.5 times IQR from the first or third quartiles. *GBR = Great-Britain; NLD = Netherlands*

**Supplementary Tables**

**Supplementary Table 2: Characteristics of the study population according to their country of residence.** P-values associated to either Mann-Whitney test (for age, Bristol stool scale and MGS richness) or Chi-squared test (for sex) are displayed. Values for quantitative variables are displayed as mean ± standard deviation. *GBR = Great-Britain; NLD = Netherlands*

|  | GBR | NLD | p |
| --- | --- | --- | --- |
| number | 23 | 11 | - |
| Age (years) | 40 ± 12 | 30 ± 12 | **0.032** |
| Sex (Female %) | 30 | 64 | 0.14 |
| Bristol STOOL scale | 5.8 ± 0.77 | 4.8 ± 1.3 | **0.031** |
| MGS richness | 210 ± 48 | 230 ± 43 | 0.67 |
